# Determination of Chloramphenicol in Honey Using Salting-Out Assisted Liquid-Liquid Extraction Coupled with Liquid Chromatography-Tandem Mass Spectrometry and Validation According to 2002/657 European Commission Decision

**DOI:** 10.3390/molecules25153481

**Published:** 2020-07-31

**Authors:** Serena Rizzo, Mariateresa Russo, Massimo Labra, Luca Campone, Luca Rastrelli

**Affiliations:** 1 Dipartimento di Farmacia, University of Salerno, Via Giovanni Paolo II, 84084 Fisciano (SA), Italy; srizzo@unisa.it (S.R.); rastrelli@unisa.it (L.R.); 2 Dipartimento di Agraria, Food Chemistry, Safety and Sensoromic Laboratory (FoCuSS Lab), University Mediterranea of Reggio Calabria, Via Melissari, 89124 Reggio Calabria (RC), Italy; mariateresa.russo@unirc.it; 3 Department of Biotechnology and Biosciences, University of Milano-Bicocca, Piazza Della Scienza, 20126 Milano, Italy; massimo.labra@unimib.it

**Keywords:** honey, chloramphenicol, salting-out assisted liquid-liquid extraction, Commission Decision 2002/657/EC, experimental design optimization

## Abstract

Honey is a natural food widely consumed due to its high content in nutrients and bioactive substances. In order to prevent hive infections, xenobiotics such as pesticides and antibiotics are commonly used. Chloramphenicol (CAP) is a broad-spectrum antibiotic used to treat honeybee larvae diseases. However, CAP has toxic and nondose-dependent effects in sensitive subjects; for this reason, its use has been prohibited in food-producing animals, such as the honeybee. In this study, we proposed a rapid, simple, and cheap analytical method, based on salting-out assisted liquid-liquid extraction coupled with UHPLC MS/MS detection for the accurate determination of CAP in honey to be used in routine analyses. The parameters that influence the extraction efficiency have been optimized using an experimental design in order to maximize the recovery of the analyte by reducing the matrix effects. Therefore, the developed method was internally validated according to the 2002/657/EC Decision guidelines and applied to the analysis of 96 honey samples.

## 1. Introduction

Honey is the natural sweet substance produced by honeybee (*Apis mellifera*) from the nectar of plants. The chemical composition of honey is quite variable, as it depends on both the floral source from which the bees derive the nectar and on other factors, such as seasonal and environmental changes, as well as the manufacturing processes. Honey is a functional food of wide consumption particularly rich in nutritional and bioactive substances. It contains about 200 substances, including mainly sugars (75–80%), water (16–18%), and other substances such as proteins, organic acids, vitamins, minerals, pigments, phenolic compounds, and large quantities of volatile substances [1,2]. Despite its biological and therapeutic activities, honey is not free of contaminants, mainly antibiotics, whose use in beekeeping is necessary for the treatment of bacterial infections affecting hives [3]. The most common infectious diseases affecting honeybee larvae are American and European foulbrood. American foulbrood is an infectious disease caused by the sporogenic bacterium *Paenibacillus larvae,* while European foulbrood is caused by the gram-positive *Melissococcus plutonius* [4]. Among veterinary antibiotics, chloramphenicol has been widely used [5]. Chloramphenicol (CAP) is a bacteriostatic antibiotic with a broad-spectrum of antibacterial activity, belonging to the amphenicol family. It is a strong inhibitor of bacterial protein synthesis of most gram-positive and gram-negative bacteria [6]. For this reason, as well as for its easy availability and low-cost, CAP has been widely used in beekeeping for the treatment of bacterial infections affecting hives. Unfortunately, its incorrect use has allowed it to be found as a residue in honey. In addition to bacteria, the inhibiting action of protein synthesis is exercised at the mitochondrial level of mammalian cells: Immature cells in active proliferation (erythropoietic bone marrow cells) are particularly sensitive [7]. Due to its serious side effects, such as severe forms of agranulocytosis and aplastic anemia, the International Agency for Research on Cancer (IARC) included CAP in group 2A (potentially carcinogenic molecules for humans). Consequently, regulatory agencies have established strict rules regarding the use of CAP in food in order to reduce public health problems. Due to its high toxicity and side effects on human beings and animals, the use of CAP was banned both in European Union (Reg. 2010/37/UE) [8] and in other countries of the world including USA, China, and Brazil. Furthermore, the European Commission set the Minimum Required Performance Limit (MRPL) at 0.3 µg/kg for CAP confirmatory methods in all food from animal origin (Dec. 2003/181/EC). The MRPL represents the minimum analyte concentration that a method is able to determine and confirm, accounting for laboratory performance [9]. Therefore, developing reliable and sensitive analytical methods became an important issue to accurately determine CAP trace levels in honey samples. Nowadays, we tend to divide the analytical methods in screening methods, often utilizing immunoassays, and confirmatory methods based on gas chromatography-mass spectrometry (GC-MS) or liquid chromatography–tandem mass spectrometry (LC-MS/MS) [10,11,12]. According to 2002/657/EC Decision, screening methods are used in short time analyses of a large number of samples in order to search for potential noncompliant results; confirmatory methods, such as the proposed method, provide complete information on the chemical structure capable of uniquely identifying and quantifying the substance of interest [13]. Furthermore, in order to reduce the matrix effect for trace analysis, sample pretreatment is usually necessary in quantitative determination of target analytes in complex matrices. Liquid-liquid extraction (LLE) [14], solid-phase extraction (SPE) [15], matrix solid-phase dispersion (MSPD) [16], and molecular imprinted polymer (MIP) extraction [17] have been utilized for sample pretreatment prior to quantitative determination of CAP in various food matrices. Although capable of removing most of the matrix interferences, these techniques are lengthy, laborious, and poorly eco-friendly due to the extensive use of organic solvents. In the last few years, significant advantages have been made in order to make sample pretreatments easier, faster, and more effective [18,19,20,21,22]. In this perspective, the interest on salting-out assisted liquid-liquid extraction (SALLE) as an unconventional sample preparation method has significantly increased [23]. The salting-out phenomenon consists of adding an electrolyte to an aqueous solution to increase a distribution ratio of a solute and has been widely used for different purposes [24]. An interesting application of the salting-out process is the use of a water-miscible organic solvent as extractant, resulting in the formation of a biphasic system. Basically, the addition of an electrolyte (or electrolyte mixture) allows the weakening or the disruption of the solvation forces between the organic solutes and the aqueous solvent in favor of the organic solvent [25,26]. In this study, acetonitrile was used as extracting solvent because its polarity permits the extraction of a wide range of compounds and its toxicity, which is lower than the conventional liquid–liquid extraction solvents, makes it more suitable within a green chemistry context. The optimization of the extraction parameters was performed by an experimental design, in order to discover the experimental conditions producing the best possible analytical performance. The multivariate approach obtained from the experimental design allows numerous advantages compared to the univariate approach knows as one variable at a time (OVAT). Some of these are the possibility to study the interactions between factors and the nonlinear relations with the responses, the possibility to find the absolute optimal conditions’ extraction in the studied domain, and the reduction of the number of experiments, time, costs, and efforts during the analysis [27]. So, the aim of this work was the development of a salting-out assisted liquid-liquid extraction method coupled with liquid chromatography-tandem mass spectrometry (SALLE-LC-MS/MS) in order to identify and confirm CAP in honey and finally the validation of the developed method along the guidelines given in 2002/657/EC [13].

## 2. Results and Discussion

The use of veterinary drugs in livestock production is inevitable as they are essential for disease treatment, disease prevention, and productivity improvement. However, the large use of these drugs can lead to the accumulation of their residue in foods and environment with negative consequences on human health, such as antibiotic resistance and allergies. In recent years, the risk arising from consuming contaminated foodstuffs of animal origin caused great concern among scientists, food experts, and informed consumers. Therefore, the development of rapid analytical methods plays an important role on the increase of samples’ throughput to assess food safety. These analytical procedures must satisfy the requirements of 2002/657/EC Decision, which provides strict rules and specifies common criteria for the interpretation of results.

### 2.1. Optimization of SALLE by Experimental Design

The complex composition of honey samples and the low MRPL established for CAP impose the use of an efficient samples’ treatment to operators, in order to remove matrix interferences and preconcentrate CAP prior to its determination. A careful optimization is necessary to select the best extraction condition; this goal was achieved by carrying out a factorial experimental design. The influence of four independent variables such as honey-diluted volume (HDV, 3–5 mL), extraction solvent volume (ACN, 2–4 mL), pH (2–12), and salting-out percentage (NaCl, 15–25%) were simultaneously evaluated taking into account three response factors: Supernatant volume (SV, mL), extraction recovery (ER, %), and normalized matrix effect expressed as percentage of suppression (nME, %), particularly, SV as variable to minimize, ER% as variable to maximize, and nME% as hit target equal to 0. Recoveries were calculated by comparing pre-spiked with post-spiked extraction at the same concentration levels, in order to reduce the contribution of matrix effects; nME was determined by comparing post-spiked extraction with the reference standard at the same concentration levels. The experimental results of the screening design are shown in Table S1. In order to determine factors that may statistically influence each variable, the analysis of variance (ANOVA) was carried out and the results are summarized in Table 1.

**Figure 1:**
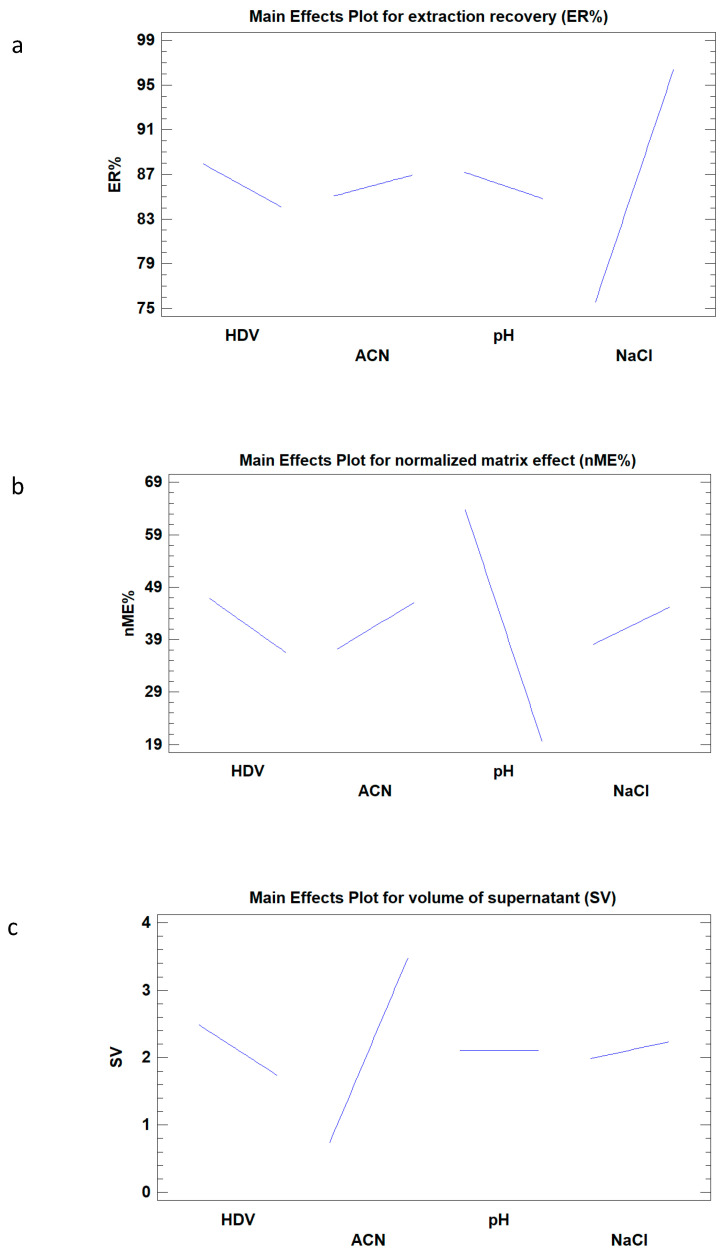
Main effect plot of the three response factors: (**a**) Extraction recovery (ER%), (**b**) normalized matrix effect (nME), and (**c**) supernatant volume (SV), on the four controllable variables: (HDV) honey-diluted volume (mL), extraction solvent volume (ACN), pH, and NaCl percentage of the honey solution.

In the ANOVA table, *p*-value less than 0.05 indicate the statistical significance of an effect at 95% confidence level. Furthermore, as can be clearly seen, the recovered volume of the extraction solvent (ACN) was influenced by both solvent extraction volume and honey dilution volume (*p*-value < 0.05) while ER and nME were statistically influenced by pH and NaCl percentage of honey-diluted solution, respectively. Regarding the interaction among the four variables, no significant interactions were found (*p*-value > 0.05). Therefore, their *p*-values were not reported in the Table 1. The main effects’ plot (Figure 1) shows that the slope of the line is proportional to the size of the effect and that the direction of the line has a positive or negative influence of each effect on each variable. As shown in the main effect plot of ER (Figure 1a), the NaCl percentage in honey solution linearly increases the recovery of CAP, while the other three effects play a slight influence on the variable. The main effect plot of nME (Figure 1b) shows that pH has a strong influence because the suppression of CAP electro spray ionization source increases when acidic or neutral pH values were reached. This behavior may be attributed to the presence of ionizable interferences into the matrix, which could be extracted in a larger amount at low pH values. As a confirmation, after the evaporation of the extraction solvent deriving from the analysis of samples at low pH values (<7), a clearly visible yellow residue was observed. As far as the recovered SV is concerned (Figure 1c), a linear increase was observed when the ACN volume was increased, while a low amount of supernatant volume was obtained when HDV was increased.

Finally, chemometric analysis indicated that the best SALLE conditions that can eliminate the matrix effect, reduce the supernatant volume, and maximize the extraction recovery are the following: 1 g of honey diluted in 5 mL of aqueous solution with a pH value of 12 and a NaCl percentage of 25%, and a volume of 2 mL of ACN as extraction solvent (degree of desirability 79%). Before using these conditions throughout the validation procedure, preliminary evaluations of matrix effect and recoveries were carried out to verify the extraction efficiency and the effective elimination of the MS signal suppression. Extraction efficiency was assessed by calculating the recoveries of fortified honey samples (acacia, chestnuts, and citrus) at 0.3 µg/kg. The recoveries were calculated by interpolating the peak areas of ^35^Cl-CAP ion transitions (*m*/*z* precursor/product 321→257 and 321→194) in the calibration curves in solvent of the respective ions. As shown in Figure S1, good recoveries (>90%) were obtained for the three analyzed honey samples. The signal suppression (or enhancement) phenomenon, deriving from the co-eluted matrix interferences, was assessed by extracting three blank samples from different botanical origins in the optimized conditions and spiking them with CAP WSs before the evaporation of the ACN volume. CAP concentration levels of 0.15, 0.3, and 0.6 µg kg^−1^ were compared to standard working solutions at the same levels. The peak area response for both the MRM transitions of post-spiked extraction samples were comparable to those obtained in solvent (H_2_O/MeOH 70:30 *v*/*v*); this confirmed the absence of matrix effects (Figure S2).

### 2.2. Validation Protocol

The analytical methods developed to monitor residues of certain substances in food of animal origin must comply with the guidelines provided by 2002/657/CE Decision. The main purpose of this regulation, implementing the 96/23/EC Council Directive concerning the performance of analytical methods and the interpretation of the results, is to detect the illegal use of substances not allowed in the production of food from animal origin as well as to detect the improper use of unauthorized veterinary medicines in the same food products. The 96/23/EC Directive established measures that require European Member States to monitor the substances and groups of residues listed in Annex I of the Directive. These substances are grouped in two main categories: (1) *Allowed substances*, for which a maximum residue limit (MRL) was established, and (2) *prohibited substances*, for which no MRL could be established. For the last one, the European Commission set the minimum required performance limit (MRPL) for CAP at 0.3 µg/kg. Taking these assumptions into account, the proposed quantitative confirmatory method was validated according to the EC Directive.

#### 2.2.1. Liquid Chromatography and Mass Spectrometry Parameters

According to the 2002/657/EC Decision guidelines, liquid chromatography techniques coupled with mass spectrometry detection (LC-MS) are considered adequate for quantitative analysis of organic contaminants in food for human consumption, as long as a sufficient number of identification points (IPs) were met. However, at least four identification points are required for substances belonging to group 2 (*prohibited substances*) of Commission Regulation 2010/37/UE, in which CAP is included. In our case, this requirement was fulfilled by coupling liquid chromatography-tandem mass spectrometry (LC-MS/MS) and by selecting one precursor ion and two product ions (321→257; 194). The two diagnostic ions were selected during the optimization of MS and MS/MS parameters by infusing CAP standard solution at 5 µg/mL in H_2_O/MeOH (50:50 *v*/*v*). Mass spectrum of ESI in negative mode shows the typical isotopic pattern of CAP due to the two Cl-atoms in the molecule (Figure S3). According to theoretical CAP spectrum (Figure S3b), the measured abundances show that the ion at *m*/*z* 321, relative to [M−H]^−35^Cl-atoms, is the most abundant while that having *m*/*z* 323, relative to [M−H]^−37^Cl-atoms is the least. The ESI-MS/MS spectrum derived from the fragmentation of ^35^Cl-CAP (*m*/*z* 321), produced a base peak at *m*/*z* 257, chosen as quantitative ion, and the product ion at *m*/*z* 194, chosen as confirmatory ion. The presence of CAP in a sample would be characterized by comparing two parameters: (1) The relative retention time of the suspect peak and (2) the ratio of the relative abundance of its main diagnostic ions with those obtained by analysis of a positive control (QC). In particular, the relative abundance of diagnostic ions (with a relative abundance >50% of the base peak) must not differ by more than ±20% from those observed in the QC. The result of tolerance ion ratio (CV_IR_) for selected ions 194/257 in different honey matrices satisfies the permitted tolerance given by 2002/657/EC guidelines (Table 2).

**Figure 2:**
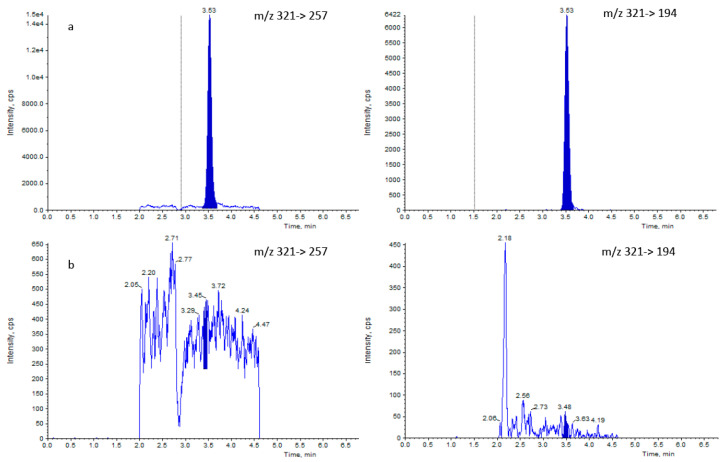
Salting-out assisted liquid-liquid extraction chromatograms for both selected transitions of spiked honey sample at MPRL (0.3 μg/kg) (**a**) and noncontaminated honey samples (**b**).

After the selection of the diagnostic ions, the LC-MS method was developed; the total time of the analysis was 12 min with an analyte retention time of 3.49 ± 0.4 (Figure 2a). This value was in agreement with the 2002/657/EC guidelines, which established that the minimum acceptable retention time for the analyte should be twice the retention time, corresponding to the void volume of the UHPLC column (0.2 min), and that the relative retention time of the analyte in matrix shall correspond to that of the calibration solution with a tolerance of ±2.5%. Furthermore, the selectivity of the method has been evaluated. According to the 2002/657/EC Decision, the selectivity is defined as the capability of the analytical method to discriminate between the target analyte molecules and isomers, degradation products, matrix interferences, and closely related substances. Selectivity has been evaluated analyzing 20 representative blank samples and checking if any interferences were noticed in the retention time region of the analyte. The chromatograms show that no interferences were eluted near the retention time of all analyzed blank samples (Figure 2b).

In addition, the analysis of the 20 blank samples allowed us to calculate the average (µ_N_) and the standard deviation (*σ_N_*) of the noise of the signal amplitude, which were used in the *CCα* and *CCβ* determination. The calculated values were µ_N_ of 235.2 and *σ_N_* of 1902.0 for precursor/product 321→257, while µ_N_ of 165.3 and *σ_N_* of 86.1 for precursor/product 321→194.

#### 2.2.2. Calibration Curves and Linearity

**Figure 3:**
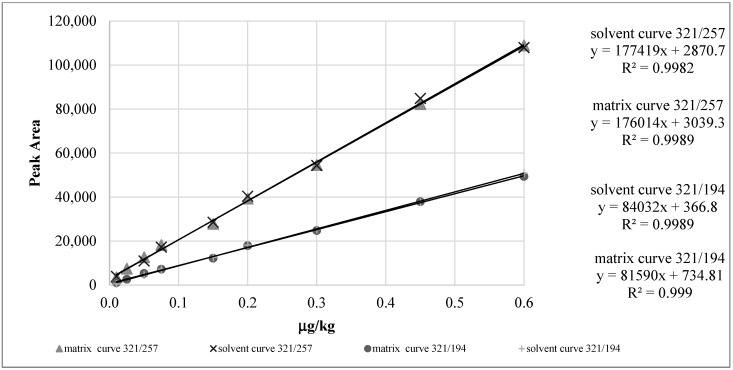
Comparison between calibration curve in solvent and in matrix at the same concentration range for 321→257 and 321→194 ion transitions.

The guidelines establish that the linearity of the analyte confirmatory methods, expressed as regression coefficient (R^2^), must be greater than 0.9980. Calibration curves were constructed by comparing the responses given by samples in solvent and in matrix at nine levels (≥ 5 + i; as suggested by guidelines) in the range of 0.01–0.6 µg/kg, considering µ_N_ as intercept. Good linearity was obtained for calibration curve in solvent (α = 177419; R^2^ = 0.9982) and calibration curve in matrix (α = 176014; R^2^ = 0.9989) relative to *m*/*z* 321→257. The same good linearity was obtained for both the calibration curves in solvent (α = 84032; R^2^ = 0.9989) and in matrix (α = 81590; R^2^ = 0.9990) relative to *m/z* 321→194. The regression analysis, performed on the two pairs of curves (calibration curves in solvent and in matrix), provides a *p*-value of 1.13E-06 for 321/257 ion and a *p*-value of 3.2 × 10^−7^ for 321/194 ion. A *p*-value < 0.05 for both curves indicates that there is no statistical difference between the calibration curves in solvent and in matrix (Figure 3), with a confidence level of 95%. Therefore, the quantification of CAP in honey samples was performed by using calibration curve in solvent.

#### 2.2.3. Recovery, Repeatability (within-Laboratory Reproducibility) and Accuracy

The repeatability was evaluated by the analysis of fortified honey samples at CAP concentrations of 0.1, 0.3, 0.45, and 0.6 µg/kg (including six replicates for each level), and carried out by two laboratory operators within three months. The concentration detected in each fortified honey sample as well as the average (AV), the standard deviation (SD), and the coefficient of variation (CV%) are summarized in Table 3.

The results show that the selected transitions provided comparable results for both recovery (%) and repeatability (SD). Since no certified reference materials (CRMs) were available in our laboratory, accuracy was assessed through the recovery of known amounts of CAP into blank samples. Three different botanical honey samples were fortified at 0.3 µg/kg and recoveries were calculated by interpolating the peak areas of ^35^Cl-CAP ion transitions (*m*/*z* precursor/product 321→257 and 321→194) in solvent calibration curves of respective ions. The calculated CV% provided an estimate of the precision of the analysis (Table 3). All data, corrected for the average of the recovery, fall within the ranges established in the 2002/657/EC guidelines; that is, the experimentally calculated concentration range must fall between −50% and +20% for MRPL ≤1 µg/kg and lowest possible CV% for concentrations <100 µg/kg.

#### 2.2.4. Decision Limit (*CCα*) and Detection Capability (*CCβ*)

Undoubtedly, the most important changes that the 96/23/EC Directive introduced into the validation protocol of analytical methods were the introduction of the decision limit (*CCα*) and the detection capability (*CCβ*) criteria. The practical meaning of these two parameters can be summarized as follow: Signals less or equal to *CCα* are considered as noise (compliant samples), signals equal or higher to *CCβ* are considered as produced from forbidden substances (noncompliant samples), and signals between these two values require further investigation to take a decision. The determination of *CCα* and *CCβ* was carried out following the guidelines proposed by Antignac et al. [28] whereby a total number of 40 honey samples, of which 20 were blank and 20 were fortified samples, at six concentration levels were analyzed. The *CCα* and *CCβ* values for both the ion transitions were calculated using Equations (1) and (3), respectively. The average of the noise (µ_N_) and the slope (*α*) of the matrix-matched calibration curve allowed us to calculate the decision limit (*CCα*). A concentration level that produced a signal-to-noise ratio of approximately 6 (0.01 µg/kg), during the analysis of the matrix-matched calibration curve, was chosen in order to calculate the detection capability (*CCβ*) through the standard deviation (σ_S_) and the coefficient of variation (*CV_S_*) of the signal (six replicates). Following the above procedure, the calculated values of *CCα* corresponded to 0.0025 µg/kg for *m*/*z* precursor/product 321→257 and 0.0052 µg/kg for *m*/*z* precursor/product 321→196. Therefore, the *CCα* calculated concentration allowed us to calculate the *CCβ* value, which corresponds to 0.0029 µg/kg for *m*/*z* precursor/product 321→257 and 0.0076 µg/kg for *m*/*z* precursor/product 321→194.

### 2.3. Real Samples Analysis

The developed analytical method was applied to the analysis of 96 honey samples from different botanical and geographical origins. A total number of 13 contaminated honey samples emerged from the analysis, of which one was purchased in a supermarket, one supplied by a local beekeeper, and 11 imported from intra- and extra-European countries. The contaminated honey samples contained CAP levels lower than 0.009 µg/kg but still higher than the detection capability (*CCβ*) and were therefore, considered noncompliant. These results confirmed the need to increase the monitoring programs of honey samples in order to guarantee the maximum quality and safety for consumers.

## 3. Material and Methods

### 3.1. Chemicals

Chloramphenicol standard (CAP) purity ≥ 98% was purchased from Sigma-Aldrich (Milan, Italy). Ultrapure water (18MΩ) was obtained through a Milli-Q system from Millipore (Bedford, MA, USA). Analytical-grade acetonitrile (ACN), methanol (MeOH), hydrochloric acid 37% *w*/*v* (HCl), sodium hydroxide (NaOH), and sodium chloride (NaCl) were purchased from Sigma-Aldrich (Milan, Italy). mass spectrometry grade water (H2O) and methanol (MeOH) were supplied by Romil (Cambridge, UK).

### 3.2. Samples and Standard Solutions

Honey samples from different botanical origin were supplied by beekeepers or purchased in supermarkets in Campania (Italy). Stock standard solution of CAP at 1000 µg/mL was prepared in acetonitrile and stored in glass vial at −18 °C for no more than one month; an intermediate standard solution at 10 µg/mL, obtained by dilution of the stock standard solution, was prepared weekly. Working standard solutions 1 and 2 (WS1 and WS2) at 100 ng/mL and 1 ng/mL, respectively, obtained by dilution of the intermediate standard solution, were prepared daily and used for sample spiking and preparation of calibration curves, in solvent and in matrix.

### 3.3. Salting-Out Assisted Liquid-Liquid Extraction Procedure

The salting-out assisted liquid-liquid extraction (SALLE), under optimized conditions, was performed on 1 g of honey, weighed into a 15-mL polypropylene centrifuge tube and diluted with 5 mL of water, previously brought to pH value of 12 (adjusted with 0.1 M NaOH) and to a concentration of 25% NaCl *w*/*v*. Afterwards, the mixture was vortexed until a homogeneous solution was obtained. Samples were extracted with 2 mL of ACN, then vortexed for 2 min in order to disperse extraction solvent into the aqueous sample solution ensuring analyte extraction. The mixture was centrifugated at 13,000 rpm for 5 min to achieve the separation between aqueous and organic phases. In order to facilitate the recovery of the extraction solvent (upper phase), water (lower phase) was removed using a Pasteur pipette. The extraction solvent (1.5 mL), containing the analyte, was transferred into a clean tube and evaporated under a gentle flow of nitrogen. Finally, the dry residue was reconstituted with 100 µL of H_2_O/MeOH (70:30 *v*/*v*), transferred into an HPLC vial and analyzed by LC-MS/MS.

### 3.4. UHPLC-MS/MS Analysis

UHPLC analyses were performed using a Shimadzu Nexera X2 UHPLC system (Shimadzu, Milan, Italy) coupled with a Qtrap 6500 mass spectrometer (AB Sciex, Milan, Italy) equipped with a TurboV ion source. Analyst software (Version 1.6, (AB SCIEX™, Foster City, CA, USA)) was used for instrument control, data acquisition, and analysis. A Kinetex C18 column (50 × 2.1 mm, 1.7 µm; Phenomenex, Bologna, Italy) was used to separate the matrix components from the analyte, at a flow rate of 0.4 mL/min and a temperature of 30 °C. The mobile phase was a binary gradient of H_2_O (A) and MeOH (B). After injection (10 µL), CAP was eluted using the following gradient: 0–0.5 min, 5% B; 0.5–1.0 min, linear increase to 20% B; 1.0–3.0 min, linear increase to 40% B, hold of 1.0 min; 4.0–7.0 min, linear increase to 98% B. After each run, the column was washed (98% B, 6 min) and re-equilibrated (5% B, 6 min). The mass spectrometer operated in negative ionization mode. Nitrogen was used as nebulizer gas, heater gas, curtain gas, and collision gas. The MS and MS/MS parameters were optimized by infusing a CAP standard solution of 5 µg/mL with a flow rate of 5 µL/min. The optimized ion source parameters were: Ion spray voltage (IS)—4500 V, source temperature (TEM) 400 °C, nebulizer gas (GS1) 45 psi, heater gas (GS2) 30 psi, curtain gas (CUR) 35 psi, collision gas (CAD) in “medium” mode, entrance potential (EP)—10 V, cell exit potential (CXP)—10 V, and declustering potential (DP)—110 V. Two multiple reaction monitoring (MRM) transitions of CAP were monitored; in particular, the MRM transition ^35^Cl-CAP, *m*/*z* precursor/product transitions 321→257 (collision energy [CE]−14) was used for the quantification, while ^35^Cl-CAP, *m*/*z* precursor/product transitions 321→194 (CE—10) was used for the identification.

### 3.5. Experimental Design

In order to find the best SALLE conditions, a chemometric approach was employed. For this purpose, a 2^4^-factorial design of 18 randomized experimental runs (1 block, 16 factorial design runs, 2 centerpoints) with 7 degrees of freedom was used. In the planning of the experimental design, the influence of four independent variables at low, medium, and high level was valued. The variables evaluated were: Honey-diluted volume (HDV, mL) of 3, 4, and 5 mL; extraction solvent volume (ACN, mL) of 2, 3, and 4 mL; pH value of 2, 7, and 12, and salting-out percentage (NaCl%) of 15, 20, and 25%. The three response factors considered were: Supernatant volume (SV, mL), extraction recovery (ER, %), and normalized matrix effect expressed as percentage of suppression (nME%). In order to eliminate the influence of matrix effect on the analyte response, extraction recoveries were calculated by comparing the CAP peak area resulting from the pre-spiked extraction procedure with that resulting from the post-spiked extraction procedure. The design was performed on 1 g of honey, spiked with 30 µL of WS1 (100 ng/mL), and corresponding to 0.3 µg/kg of CAP. The range of each factor used was selected by preliminary experiments. The experimental design conditions and the response factors results are shown in Table S1. Analysis of variance (ANOVA), obtained from the fitted model, was carried out to determine the statistical significance of the experimental variables and the optimum experimental conditions that minimized SV, maximized ER%, and hit the target of 0 for nME%. The experimental design was set up using the Statgraphics Centurion XVI software, Version 16.1 (Rockville, MD, USA).

### 3.6. Matrix Effect Evaluation

The matrix effect phenomena (signal suppression or enhancement) was evaluated through the analysis of blank honey samples from different botanical origins (acacia, chestnut, and citrus), under optimized conditions. After SALLE procedure, the extracts and the solvent solutions (H_2_O/MeOH 70:30 *v*/*v*) were spiked with an appropriate volume of CAP WS2 at three concentration levels (0.1, 0.3, and 0.6 μg/mL). Then, samples were evaporated under nitrogen, reconstituted in 100 μL of H_2_O/MeOH 70:30 *v*/*v,* and injected into the UHPLC-MS/MS system. The analyte peak area of the honey extracts and the solvent sample solutions were compared to evaluate the contribution of matrix interferences on the response of ion transitions.

### 3.7. Method Validation

The calibration curves in solvent and in matrix were constructed by setting the peak area of each MRM transition as a function of analyte concentration (nine levels), ranging from 0.01 to 0.6 µg/kg corresponding to 0.1–6 µg/L. The matrix-matched calibration curve was made by spiking a mixture of the three uncontaminated honey samples with an appropriate addition of WS volume, in order to obtain the following concentrations: 0.01, 0.025, 0.05, 0.075, 0.15, 0.20, 0.30, 0.45, and 0.60 µg/kg. The calibration curve in solvent was made in the same concentration range by diluting pure standards of CAP into H_2_O/MeOH (70:30 *v*/*v*). For both curves, the intercept, the standard deviation, and the coefficient of variation were calculated. The quantification of CAP in honey samples was carried out using the calibration curve of the 321→257 transition, whereas the 321→194 transition was used for the analyte confirmation. Linearity and sensitivity were evaluated through the regression coefficient (R^2^) and the slope (*α*) of the calibration curve, respectively. The ruggedness and the specificity of the method were evaluated through the average (µ_N_) and standard deviation (*σ_N_*) of the noise amplitude of the blank samples; µ_N_ was also used as intercept in the calibration curve. Then, *σ_N_* and *α* permitted us to calculate the decision limit (*CCα*) by the Equation (1):

(1) $$CC\alpha =\frac{2.33\;\sigma_{N}}{\alpha }$$

The repeatability was evaluated through the standard deviation of the signal amplitude (*σ_S_*) produced by the analysis of samples at a CAP concentration inducing a signal-to-noise ratio approximately equal to 6. Finally, *σ_N_*, *α*, and the coefficient of variation of the signal amplitude (*CV_S_*) permitted us to calculate the detection capability (*CCβ*) by the Equation (2):

(2) $$CC\beta =\frac{2.33\;\sigma_{N}+1.64\;\sigma_{N}\;CV_{S}}{\alpha \;\left(1-1.64\;CV_{S}\right)}$$

The use of *CV_S_* in the *CCβ* calculating formula is preferable in order to minimize the estimation error. Otherwise, *CCβ* can be calculated not taking this estimation error into account, by the Equation (3):

(3) $$CC\beta =CC\alpha +\frac{1.64\;\sigma_{S}}{\alpha }$$

In this validation protocol, *CCβ* was calculated using the Equation (3).

Accuracy was evaluated by spiking uncontaminated honey samples from different botanical origin (acacia, chestnut, and citrus) with an appropriate volume of WS2 (10 ng/mL), in order to obtain the following concentrations: 0.1, 0.3, 0.45, and 0.6 μg/kg.

## 4. Conclusions

In this study, a confirmatory method for the quantitative analysis of CAP in honey and its validation according to the 2002/657/EC Decision was proposed. The use of the SALLE procedure, during the sample preparation step, allowed the extraction of the analyte so effectively that it fully met the requirements of the guidelines. In addition, the reduced use of organic solvents and disposable materials reduced the environmental impact. The selection and the optimization of the extraction parameters through the experimental design allowed us to reveal CAP at trace levels, whereas its quantification by tandem mass spectrometry (MS/MS) provides the selective confirmation of the analyte. Furthermore, the total absence of matrix effects in the optimized extraction conditions allows us to quantify CAP with good precision and accuracy, avoiding the use of an isotopic standard. All the analytical parameters were satisfactory in terms of analyte recovery, repeatability, specificity, and ruggedness. The simplicity and the rapidity of the extraction technique as well as the sensitivity and the accuracy of the detection method are the main advantages of the proposed procedure. These features allow us to increase the productivity of the samples, reducing the use of toxic solvents and making the method both economical and eco-friendly.

## Figures and Tables

**Table 1:** Analysis of variance (ANOVA) on each factor affecting dependent variable of interest.

Factor	*p*-Value SV (mL)	*p*-Value ER (%)	*p*-Valuen ME (%)
HDV (mL)	0.0001	0.1900	0.0951
ACN (mL)	0.0001	0.5151	0.1476
pH	1.0000	0.4120	0.0003
NaCl (%)	0.0987	0.0001	0.2382

*p*-value numbers < 0.05 indicate significant factors as identified by the analysis of variance (ANOVA).

**Table 2:** The ion ratios 194/257 of [M−H]^−35^Cl-atomsand tolerance ion ratio (CV_IR_%) in honey samples from different botanical origin spiked at 0.3 µg/kg.

Botanical Origin	Ion Ratio 194/257	CV_IR_ (%)
CAP STD (QC)	42	2
Acacia honey	43	4
Chestnut honey	45	3
Citrus honey	41	12

**Table 3:** Performances of the analytical method.

	Levels (μg mL^−1^)							
	0.1	0.1	0.3	0.3	0.45	0.45	0.6	0.6
Transition (m/z)	321→257	321→194	321→257	321→194	321→257	321→194	321→257	321→194
**Acacia**								
Recovery (%) ± SD	94 ± 4.7	98 ± 9.2	95 ±1.4	92 ± 3.6	100.3 ±1.19	100.1 ± 2.7	95 ±3.3	105.1 ± 7.1
Precision (CV)	5.03	9.63	1.45	3.33	1.15	2.33	3.55	6.73
**Chestnuts**								
Recovery (%) ± SD	97 ± 6.5	96 ± 5.36	98 ± 3.3	94 ± 2.36	100.8 ± 3.5	101 ± 2.7	100.2 ± 3.3	98.8 ± 3.2
Precision (CV)	6.75	5.67	3.38	2.37	3.45	2.74	3.38	3.3
**Citrus**								
Recovery (%) ± SD	92 ± 1.4	98 ± 8.8	101 ± 2.2	98 ± 2.3	96 ± 1.4	95 ± 1.7	94 ± 4.2	95 ± 3.19
Precision (CV)	1.58	7.79	2.39	2.59	3.35	1.9	3.39	1.79

## Supplementary Materials

The following are available online, Figure S1: Evaluation of preliminary extraction recoveries on three different botanical honey under optimized experimental design conditions, Figure S2: Evaluation of preliminary matrix effects on three different botanical honey at three concentration levels under optimized experimental design conditions. Figure S3: Theoretical (a) and experimental (b) isotopic pattern of CAP in negative ion mode. Table S1: Design matrix for the 24-Factorial design and obtained result for each run.

## References

[1] Da Silva P.M., Gauche C., Gonzaga L.V., Costa A.C.O., Fett R. (2016). Honey: Chemical composition, stability and authenticity. Food Chem..

[2] Campone L., Piccinelli A.L., Pagano I., Carabetta S., Di Sanzo R., Russo M., Rastrelli L. (2014). Determination of phenolic compounds in honey using dispersive liquid-liquid microextraction. J. Chromatogr. A.

[3] Al-Waili N., Salom K., Al-Ghamdi A., Ansari M.J. (2012). Antibiotic, pesticide, and microbial contaminants of honey: Human health hazards. Sci. World J..

[4] Krongdang S., Evans J.D., Chen Y., Mookhploy W., Chantawannakul P. (2019). Comparative susceptibility and immune responses of Asian and European honey bees to the American foulbrood pathogen, Paenibacillus larvae. Insect Sci..

[5] Bargańska Z., Namieśnik J., Ślebioda M. (2011). Determination of antibiotic residues in honey. TrAC Trends Anal. Chem..

[6] Cundliffe E., McQuillen K. (1967). Bacterial protein synthesis: The effects of antibiotics. J. Mol. Biol..

[7] Watson D.H. (2004). Pesticide, Veterinary and Other Residues in Food.

[8] Commission Regulation (2010). Commission Regulation (EU) No 37/2010 of 22 December 2009 on pharmacologically active substances and their classification regarding maximum residue limits in foodstuffs of animal origin. Off. J. Eur. Union.

[9] The Commission of the European Communities (2003). Commission Decision (2003/181/EC) of 13 March 2003 amending Decision 2002/657/EC as regards the setting of minimum required performance limits (MRPLs) for certain residues in food of animal origin. Off. J. Eur. Union.

[10] Shen H.Y., Jiang H.L. (2005). Screening, determination and confirmation of chloramphenicol in seafood, meat and honey using ELISA, HPLC-UVD, GC-ECD, GC-MS-EI-SIM and GCMS-NCI-SIM methods. Anal. Chim. Acta.

[11] Barreto F., Ribeiro C., Barcellos Hoff R., Dalla Costa T. (2016). Determination of chloramphenicol, thiamphenicol, florfenicol and florfenicol amine in poultry, swine, bovine and fish by liquid chromatography-tandem mass spectrometry. J. Chromatogr. A.

[12] Xie X., Wang B., Pang M., Zhao X., Xie K., Zhang Y., Wang R., Shi H., Zhang G., Zhang T. (2018). Quantitative analysis of chloramphenicol, thiamphenicol, florfenicol and florfenicol amine in eggs via liquid chromatography-electrospray ionization tandem mass spectrometry. Food Chem..

[13] European Commission (2002). Commission Decision (2002/657/EC) of 12 August 2002 implementing Council Directive 96/23/EC concerning the performance of analytical methods and the interpretation of results. Off. J. Eur. Commun..

[14] Rodziewicz L., Zawadzka I. (2008). Rapid determination of chloramphenicol residues in milk powder by liquid chromatography-elektrospray ionization tandem mass spectrometry. Talanta.

[15] Sheridan R., Policastro B., Thomas S., Rice D. (2008). Analysis and occurrence of 14 sulfonamide antibacterials and chloramphenicol in honey by solid-phase extraction followed by LC/MS/MS analysis. J. Agric. Food Chem..

[16] Pan X.D., Wu P.G., Jiang W., Ma B.J. (2015). Determination of chloramphenicol, thiamphenicol, and florfenicol in fish muscle by matrix solid-phase dispersion extraction (MSPD) and ultra-high pressure liquid chromatography tandem mass spectrometry. Food Control.

[17] Samanidou V., Kehagia M., Kabir A., Furton K.G. (2016). Matrix molecularly imprinted mesoporous sol-gel sorbent for efficient solid-phase extraction of chloramphenicol from milk. Anal. Chim. Acta.

[18] Campone L., Celano R., Piccinelli A.L., Pagano I., Cicero N., Di Sanzo R., Carabetta S., Russo M., Rastrelli L. (2019). Ultrasound assisted dispersive liquid-liquid microextraction for fast and accurate analysis of chloramphenicol in honey. Food Res. Int..

[19] Campone L., Piccinelli A.L., Celano R., Russo M., Valdés A., Ibáñez C., Rastrelli L. (2015). A fully automated method for simultaneous determination of aflatoxins and ochratoxin A in dried fruits by pressurized liquid extraction and online solid-phase extraction cleanup coupled to ultra-high-pressure liquid chromatography-tandem mass spectrometry. Anal. Bioanal. Chem..

[20] Campone L., Piccinelli A.L., Celano R., Russo M., Rastrelli L. (2013). Rapid analysis of aflatoxin M1 in milk using dispersive liquid-liquid microextraction coupled with ultrahigh pressure liquid chromatography tandem mass spectrometry. Anal. Bioanal. Chem..

[21] Campone L., Piccinelli A.L., Celano R., Rastrelli L. (2012). PH-controlled dispersive liquid-liquid microextraction for the analysis of ionisable compounds in complex matrices: Case study of ochratoxin A in cereals. Anal. Chim. Acta.

[22] Campone L., Piccinelli A.L., Rastrelli L. (2011). Dispersive liquid-liquid microextraction combined with high-performance liquid chromatography-tandem mass spectrometry for the identification and the accurate quantification by isotope dilution assay of Ochratoxin A in wine samples. Anal. Bioanal. Chem..

[23] Valente I.M., Gonçalves L.M., Rodrigues J.A. (2013). Another glimpse over the salting-out assisted liquid-liquid extraction in acetonitrile/water mixtures. J. Chromatogr. A.

[24] Rice N.M., Irving H.M.N.H., Leonard M.A. (2007). Nomenclature for liquid-liquid distribution (solvent extraction) (IUPAC Recommendations 1993). Pure Appl. Chem..

[25] Teju E., Tadesse B., Megersa N. (2017). Salting-out-assisted liquid–liquid extraction for the preconcentration and quantitative determination of eight herbicide residues simultaneously in different water samples with high-performance liquid chromatography. Sep. Sci. Technol..

[26] Campone L., Piccinelli A.L., Celano R., Pagano I., Russo M., Rastrelli L. (2016). Rapid and automated analysis of aflatoxin M1 in milk and dairy products by online solid phase extraction coupled to ultra-high-pressure-liquid-chromatography tandem mass spectrometry. J. Chromatogr. A.

[27] Vera Candioti L., De Zan M.M., Cámara M.S., Goicoechea H.C. (2014). Experimental design and multiple response optimization. Using the desirability function in analytical methods development. Talanta.

[28] Antignac J.P., Le Bizec B., Monteau F., Andre F. (2003). Validation of analytical methods based on mass spectrometric detection according to the ‘2002/657/EC’ European decision: Guideline and application. Anal. Chim. Acta.

