# Supplementary material for: Determination of Chloramphenicol in Honey Using Salting-Out Assisted Liquid-Liquid Extraction Coupled with Liquid Chromatography-Tandem Mass Spectrometry and Validation According to 2002/657 European Commission Decision
Source: Molecules. 2020 Jul 31;25(15):3481. doi: 10.3390/molecules25153481 (PMC7435715; doi:10.3390/molecules25153481)
Supplement: Supplementary file 1 [file molecules-25-03481-s001.pdf]

Supplementary Materials for
Determination of Chloramphenicol in Honey Using Salting-Out Assisted Liquid–Liquid Extraction Coupled with Liquid Chromatography–Tandem Mass Spectrometry and Validation According To 2002/657/EC Decision

Table S1. Design matrix for the 2^4-Factorial design and obtained result for each run.

Independent variables						Dependent variables		
Block	Run	HDV mL	ACN mL	pH	NaCl %	SV mL	ER %	nME %
1	1	3	2	12	25	1.4	96	23
1	2	5	2	12	25	0.8	94	11
1	3	5	4	12	25	3.1	94	16
1	4	5	2	12	15	0.3	65	0
1	5	3	4	12	15	3.5	72	25
1	6	3	4	2	25	3.6	91	63
1	7	4	3	7	20	2.1	89	60
1	8	4	3	7	20	2.1	88	69
1	9	3	4	12	25	3.6	105	36
1	10	3	4	2	15	3.5	83	65
1	11	5	4	2	15	2.9	81	67
1	12	3	2	2	25	1.4	92	65
1	13	5	2	2	25	0.9	100	63
1	14	3	2	12	15	1.2	80	11
1	15	5	4	12	15	2.8	70	12
1	16	5	4	2	25	3.1	97	62
1	17	5	2	2	15	0.3	69	38
1	18	3	2	2	15	1.2	82	64

(HDV) Honey diluted volume, (ACN) extraction solvent volume, pH, and NaCl percentage of honey diluted solution

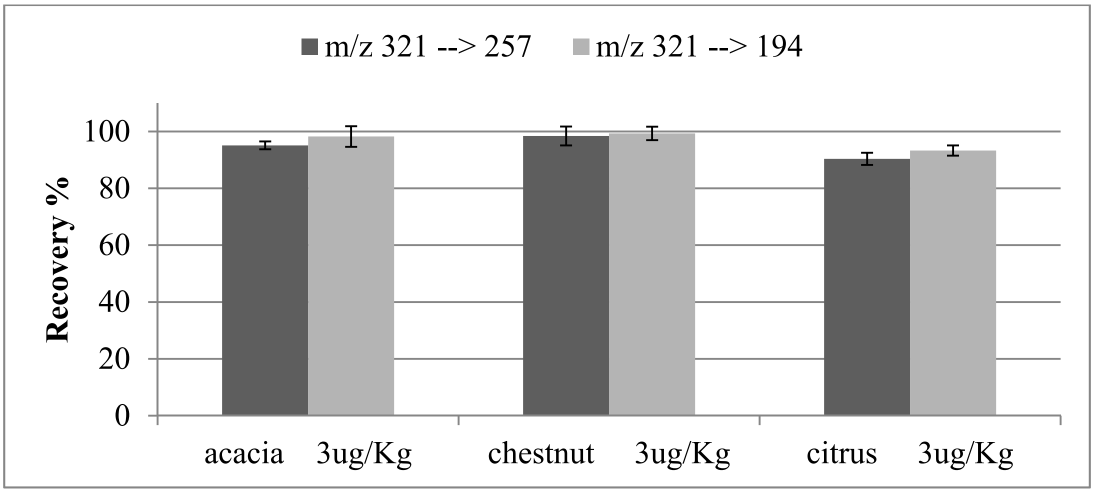

Figure S1. Evaluation of preliminary extraction recoveries on three different botanical honey under optimized experimental design conditions.

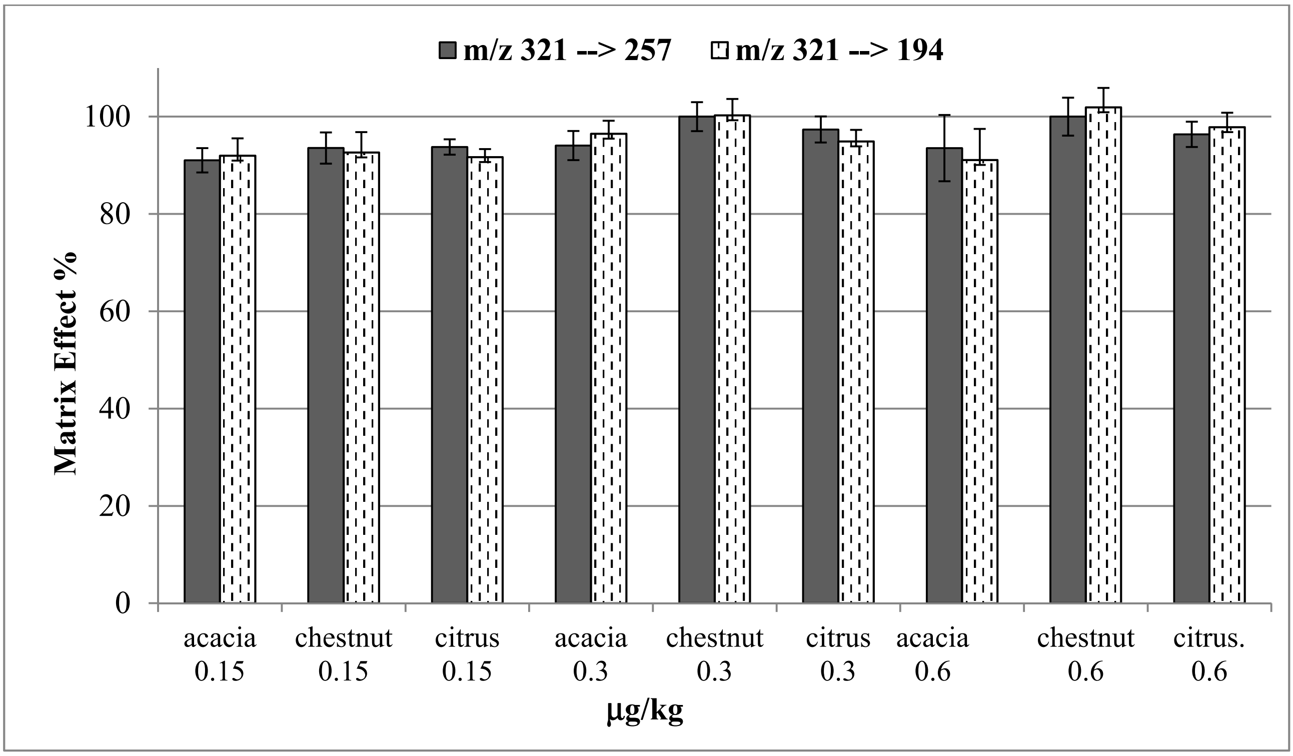

Figure S2. Evaluation of preliminary matrix effects on three different botanical honey at three concentration levels under optimized experimental design conditions.

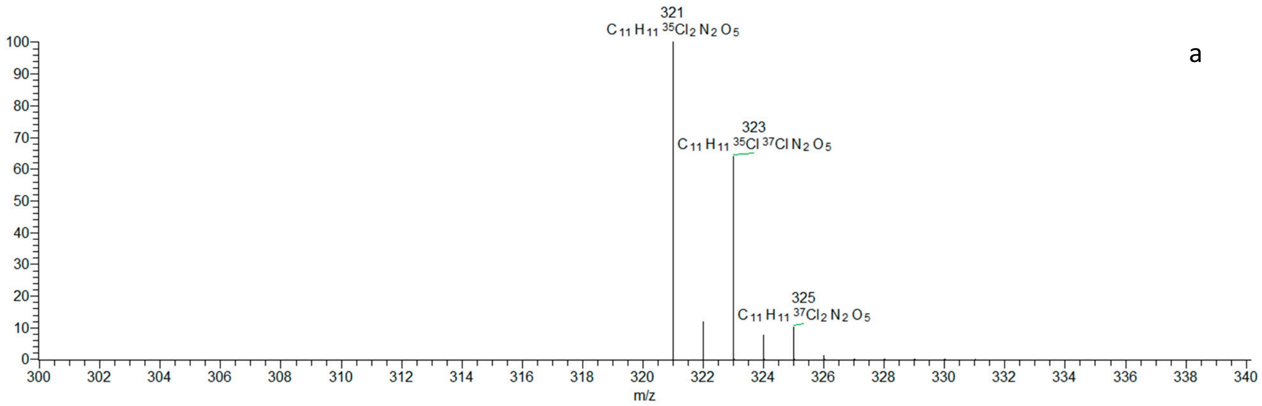

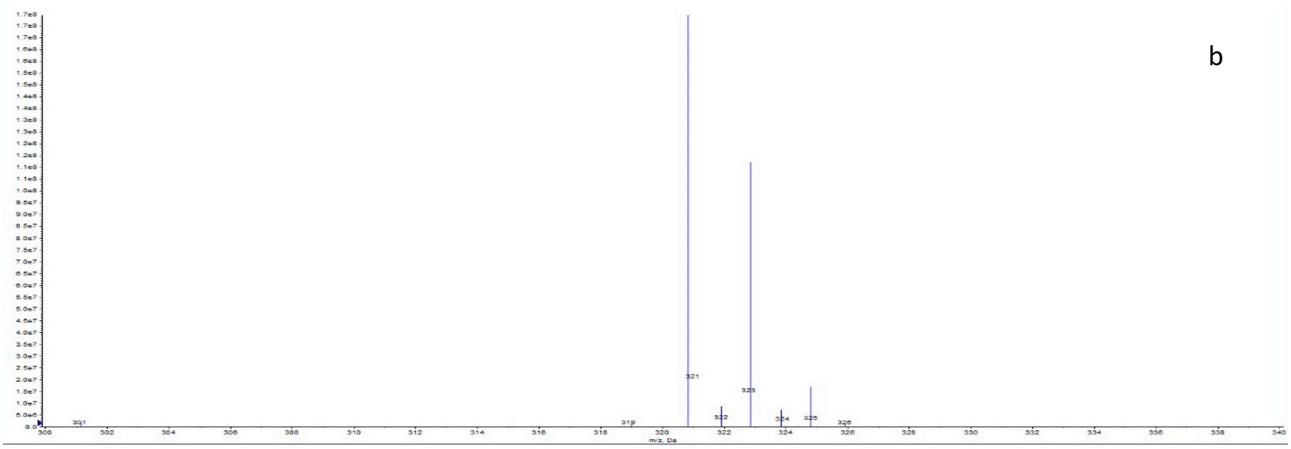

Figure S3. Theoretical (a) and experimental (b) isotopic pattern of CAP in negative ion mode.
